# *GPNMB* contributes to a vicious circle for chronic obstructive pulmonary disease

**DOI:** 10.1042/BSR20194459

**Published:** 2020-06-19

**Authors:** Xi-Juan Zhang, Zhong-Hua Cui, Yan Dong, Xiu-Wen Liang, Yan-Xin Zhao, Ancha Baranova, Hongbao Cao, Ling Wang

**Affiliations:** 1 Department of Geriatrics, First Affiliated Hospital of Soochow University, Suzhou 215006, China; 2 Department of Geriatrics, First People’s Hospital of Hulun Buir, Inner Mongolia 021008, China; 3 Department of Geriatrics, Second People’s Hospital of Lianyungang, Lianyungang, Jiangsu 222000, China; 4 School of Systems Biology, George Mason University, Fairfax, VA 22030, U.S.A.; 5 Research Centre for Medical Genetics, 1 Moskvorechie str, Moscow, Russia; 6 Department of Psychiatry, First Hospital/First Clinical Medical College of Shanxi Medical University, Taiyuan, Shanxi Province, 030001, China; 7 Boxi Clinic Center, First Affiliated Hospital of Soochow University, Suzhou 215006, China

**Keywords:** chronic obstructive pulmonary disease, Osteoporosis, mega-analysis, partial mega-analysis, pathway analysis

## Abstract

Osteoporosis (OP) is significant and debilitating comorbidity of chronic obstructive pulmonary disease (COPD). We hypothesize that genetic variance identified with OP may also play roles in COPD. We have conducted a large-scale relation data analysis to explore the genes implicated with either OP or COPD, or both. Each gene linked to OP but not to COPD was further explored in a mega-analysis and partial mega-analysis of 15 independently collected COPD RNA expression datasets, followed by gene set enrichment analysis (GSEA) and literature-based pathway analysis to explore their functional linked to COPD. A multiple linear regression (MLR) model was built to study the possible influence of sample size, population region, and study date on the gene expression data in COPD. At the first step of the analysis, we have identified 918 genes associated with COPD, 581 with OP, and a significant overlap (*P*<2.30e-140; 210 overlapped genes). Partial mega-analysis showed that, one OP gene, GPNMB presented significantly increased expression in COPD patients (*P*-value = 0.0018; log fold change = 0.83). GPNMB was enriched in multiple COPD pathways and plays roles as a gene hub formulating multiple vicious COPD pathways included gene MMP9 and MYC. GPNMB could be a novel gene that plays roles in both COPD and OP. Partial mega-analysis is valuable in identify case-specific genes for COPD.

## Introduction

Chronic obstructive pulmonary disease (COPD) is a chronic, progressive condition driven by unresolvable inflammation in the airway wall and accompanied by extensive tissue remodeling [1]. Osteoporosis is a systemic skeletal disorder recognized as one of the many extrapulmonary effects of COPD [2]. Epidemiological research shows that that prevalence of osteoporosis in COPD is higher than that in healthy elderly subjects and in some other chronic lung diseases [3,4]. A number of factors have been suggested to account for comorbid osteoporosis observed in COPD, such as confounding smoking, vitamin D deficiency, hypoxia, systemic inflammation, and various medications [4–6]. Severity of osteoporosis increased with the increase of the severity of COPD [7,8].

Both in osteoporosis and in COPD, many genes display aberrant expression patterns. It is plausible to imagine that the factors driving these expression changes may fuel both of these diseases, even though the etiology for their association remained unclear. For example, separate sets of studies analyzing the pro-inflammatory cytokines, TNF-α, IL-1β, and IL-6, which are part of the senescence-associated secretory phenotype, showed their role in deterioration of lung function in patients with COPD [9–11] and in progressive resorption of the bone [12–14]. In COPD, an increase in soluble pro-inflammatory molecules systemically tilts the balance of the osteoprotegerin (OPG)/RANKL axis toward RANKL, which propels the development of osteoporosis [15,16]. In turn, the elevated levels of RANKL provide a positive feedback to these cytokines by supporting survival of the dendritic cells that exacerbate inflammation in peripheral tissues including lungs [17], and by direct stimulation of MMP-9 [18]. This logic puts shared inflammatory milieu as the common pathogenic factor underlining both of these diseases.

Here, we explore the hypothesis that genes overexpressed in peripheral immune cells and other tissues of individuals with osteoporosis may contribute to the etiology or progression of COPD. To test this hypothesis, we compiled the lists of genes implicated in COPD and, separately, in osteoporosis, and subtracted the genes previously implicated in both of these phenotypes. In multiple independently collected COPD-related transcriptomics datasets, all genes previously identified as osteoporosis-related, but not yet highlighted by any COPD studies, were subjected to a mega-analysis of expression, followed by an enrichment analysis. We show that *GPNMB*, which encodes transmembrane protein capable of shedding its ectodomain into circulation, as a previously unrecognized factor promoting the development of COPD. We also provide preliminary evidence that *GPNMB* may be targetable either through RANKL or through MYC, or both.

## Materials and methods

In the present study, the workflow was organized as follows. First, a large-scale literature-based mining effort for COPD- and OP-related gene sets was undertaken. The overlaps of both group genes sets were studied. Then, for each gene from the list implicated in OP alone, a mega-analysis and a partial mega-analysis were conducted in 15 publicly available COPD expression datasets, which were qualified our filter standards and were retrieved from Gene Expression Omnibus (GEO) (https://www.ncbi.nlm.nih.gov/geo/). For these genes that showed significant expression change across analyzed datasets, a Gene Set Enrichment Analysis (GSEA) and a literature-based functional pathway analysis was conducted, then conclusions on their pathogenic significance in COPD were made. In addition, a multiple linear regression (MLR) model was employed to study possible influence of sample size, population region, and study date on the gene expression levels in COPD.

### Literature-based relation data

**Figure 1:**
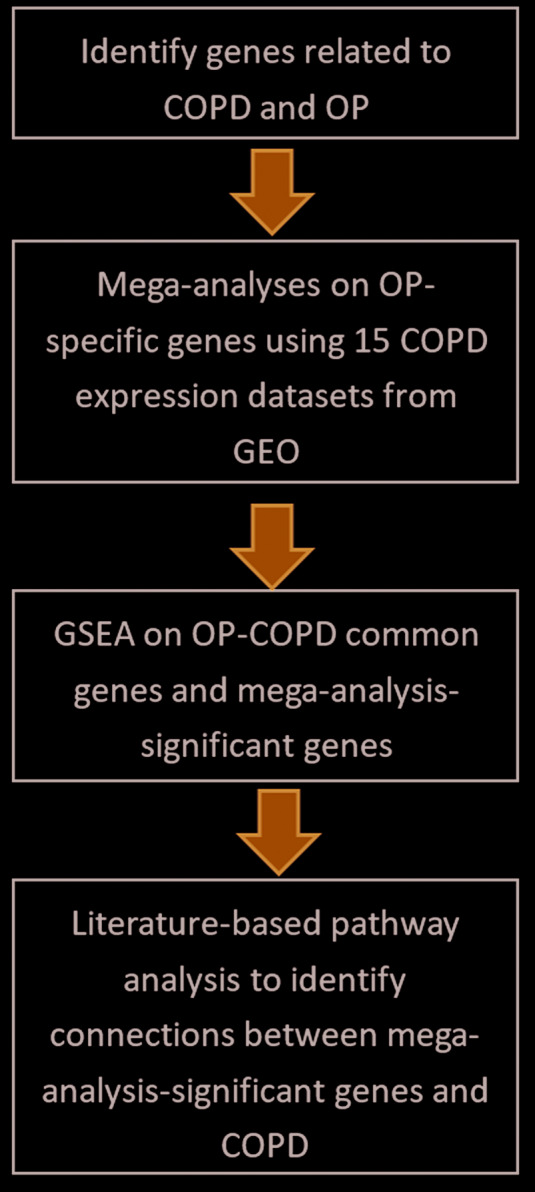
Diagram of the workflow

Relation data for both OP and COPD were extracted from existing literature and analyzed using Pathway Studio (www.pathwaystudio.com) and then were downloaded into a genetic database OP_COPD, hosted at http://database.gousinfo.com. The downloadable format of the database in excel is available at http://gousinfo.com/database/Data_Genetic/OP_COPD.xlsx. Beside the list of analyzed genes (OP_COPD→OP_Specific_Genes, COPD_Specific_Genes, and Common genes), the supporting references for each disease-gene relation are presented at database OP_COPD (OP_COPD→Ref4_OP_Specific_genes, Ref4_COPD_Specific_genes, and Ref4_Common_genes), including titles of the references and the sentences describing identified disease–gene relationships. The information could be used to locate a detailed description of an association of a candidate gene with OP and COPD. Please see Figure 1 for the workflow of this study.

### Data selection for mega-analysis

All expression datasets were searched at GEO through a keyword ‘chronic obstructive pulmonary disease’ (*N*=171). Then, we applied the following standards to do the further filter: (1) The organism is *Homo sapiens*; (2) The data type is RNA expression; (3) The sample size is no less than 10; (4) the studies are performed according to case–control design; (5) the dataset and its format files are publically available. Finally, a total of 15 datasets remained available for the mega-analysis (Table 1). The sample profile of the COPD cases and normal controls were extracted from each dataset, and other samples were excluded for analysis. For instance, the GEO dataset GSE12472 included COPD patients with/without squamous cell lung carcinoma and no COPD subjects with/without squamous cell lung carcinoma. We only used the data of COPD patients and healthy controls without squamous cell lung carcinoma, resulting in a sample size of 10/18 instead of originally 27/26, for COPD cases and healthy controls, respectively.

**Table 1:** Datasets used for COPD-osteoporosis relation mega-analysis

Study name	GEO ID	#Control/#Case	Country	Sample source
Tilley et al., 2011	GSE11784	135/22	U.S.A.	Airway epithelial cells
Raman et al., 2010	GSE11906	73/33	U.S.A.	Large airway epithelial cells
Boelens et al., 2009	GSE12472	10/18	Netherlands	normal bronchial epithelial and lung LSCC tumor cells
Shaykhiev et al., 2009	GSE13896	58/12	U.S.A.	Alveolar macrophages
Poliska et al., 2011	GSE16972	6/6	Hungary	Alveolar macrophage and PBMC
Radom-Aizik et al., 2006	GSE1786	12/12	U.S.A.	Vastus lateralis biopsy
Bosco et al., 2010	GSE19903	10/10	Australia	Sputum cells
Kalko et al., 2013	GSE27536	24/30	U.K.	Musculus vastus lateral
Kalko et al., 2014	GSE27543	6/10	U.K.	Musculus vastus lateral
Bastos et al., 2016	GSE37768	20/18	Spain	Peripheral lung tissue
Ezzie et al., 2012	GSE38974	9/23	U.S.A.	Lung tissue
Bowler et al., 2013	GSE42057	42/94	U.S.A.	PBMC
Tedrow et al., 2013	GSE47460	17/75	U.S.A.	Whole lung
Vucic et al., 2014	GSE56341	14/8	Canada	Small airway epithelia
Bhattacharya et al., 2008	GSE8581	19/16	U.S.A.	Whole lung

Abbreviation: PBMC, peripheral blood mononuclear cell.

To note, the selection of the data covers all COPD expression array datasets from GEO, which is owned by National Institute of Health (NIH of U.S.A.). The datasets are publicly available, and no permission or confirmation is needed from any individual investigators. Moreover, the dataset extraction has no selection bias concerning publication journals, owner affiliations, and authors. Besides, the original data rather than the processed results of each dataset were used to perform the analysis in the present study, which avoids possible noise caused by individual data process.

### Mega-analysis models

Both the fixed- and random-effects model [19] were employed to study the effect size of OP-related genes in case of COPD. For each expression dataset, the log fold change (LFC) was calculated for the COPD samples and used as the index of effect size in mega-analysis. The expression data were normalized and log2-transformed if not done in the original dataset. Results from both models were reported and compared. The heterogeneity of the mega-analysis was analyzed to study the variance within and between different studies. In the case that the total variance *Q* is equal to or smaller than the expected between-study variance d*f*, the statistic ISq = 100% × (*Q* − d*f*)/*Q* will be set as 0, and a fixed-effects model was selected for the mega-analysis. Otherwise, a random-effects model was selected. The *Q*–*P* represents the probability that the total variance is coming from within-study only. All analyses were conducted by an individually developed MATLAB (R2017a) mega-analysis package.

### Partial mega-analysis models

To discover genes present significance in part (e.g., 50%) of the studies/datasets but not in all datasets, we performed a partial mega-analysis, where 50% top studies/datasets were employed for the mega-analysis of a gene. We define the ‘top datasets’ for a gene as these datasets that demonstrate the bigger absolute value of effect size than the rest datasets. To note, the ‘top datasets’ for different genes could be different.

Results from both mega-analysis and partial mega-analysis were reported and compared, with significant genes identified following the criteria: *P*<0.005 and effect size (LFC) > 0.59 or < −1. When a gene presents an effect size LFC> 0.59 or < −1 in the mega-analysis, it means that the change of the expression level of the gene increased by more than 50% or decreased by more than 50%. While we present all the mega-analysis results in the OP_COPD→Mega and OP_COPD→Partial-Mega, the discussion will be focused on those genes that satisfy the significant criteria.

### Multiple linear regression analysis

A multiple linear regression analysis was employed to study the possible influence of three factors on the gene expression change: sample size, population region, and study date. *P*-values and 95% confidence interval (CI) were reported for each of the factors. The analysis was done in Matlab (R 2017a) with the ‘regress’ statistical analysis package.

### Gene set enrichment analysis (GSEA)

A GSEA has been undertaken to test the overlapped genes as well as the genes that show significance in the mega-analysis. The GSEA has been conducted using Pathway Studio. The 210 overlapped genes and the mega-analysis-significant genes were used as input, and the GSEA is testing against the Gene Ontology (GO) terms. The GSEA results were reported with enrichment p-value corrected using the original Bejnamini and Hochberg false discovery ratio (FDR) procedure [20].

### Literature-based pathway analysis

In additional to GSEA, which identify known GO terms to explore the functionality of the significant genes from the mega-analysis, a literature-based functional pathway analysis was conducted with an aim to identify potential biological linkage between COPD and the target genes. The investigation was performed using the ‘Shortest Path’ module of Pathway Studio (www.pathwaystudio.com), which identifies molecules (e.g., genes and compounds) that connecting two entities (e.g., a gene and a disease) in a directional manner. Each relationship/edge was supported by at least one reference. We presented the supporting references and the related sentences in OP_COPD→Pathway_Analysis, which could be used to evaluate the relationship identified.

## Results

### Genes linked to COPD and OP

Pathway Studio guided literature data-mining for the genes associated with osteoporosis yielded 581 genes, while similar analysis performed for COPD resulted in a list of 918 genes. Despite a significant overlap of 210 genes between OP-genes and COPD-genes (Fisher Exact *P-*value = 2.30e-140), over half of the OP-related genes (371 genes, 63.86%) have not been yet implicated in COPD. Here, the Fisher Exact *P*-values were used to evaluate the significance of the overlap of two groups of genes (https://david.ncifcrf.gov/content.jsp?file=functional_annotation.html). The full list of these OP specific genes and related supporting references are presented in OP_COPD→OP_Specific_Genes and Ref4_OP_Specific_genes, respectively.

### Mega- and partial mega-analysis results

For the partial mega-analysis, only one gene, *GPNMB*, satisfied the significance criteria (LFC = 0.83, *P-*value = 0.0018, OP_COPD→Mega). Within the Top 6 gene expression studies performed in samples collected from patients with COPD, increases of *GPNMB* expression levels averaged at 78% as observed in more than 99% of the samples. However, when all 12 expression dataset were analyzed, a magnitude of observed increases dropped to 23.22% along with a share of affected samples. The effect sizes and related statistics are shown in Table 2.

**Table 2:** Analysis of GPNMP gene expression levels in 12 GEO datasets comprises COPD samples

Significant in mega-analysis	Significant in partial mega-analysis	Model	#Study	LFC	*P*-value	ISq (%)	*P*-value–*Q*
No	No	Fixed effect	12	0.30	0.063	0	0.69
Yes	Yes	Fixed effect	6	0.83	0.0018	0	0.98

LFC: log fold change (the effect size); *P*-value represents the probability that the fold change is equal to 0. ISq = 100% × (*Q* − d*f*)/*Q* represents the percentage of between-variance over total variance; *P*-value–*Q* represents the probability that the variance is coming from within-study only.

**Figure 2:**
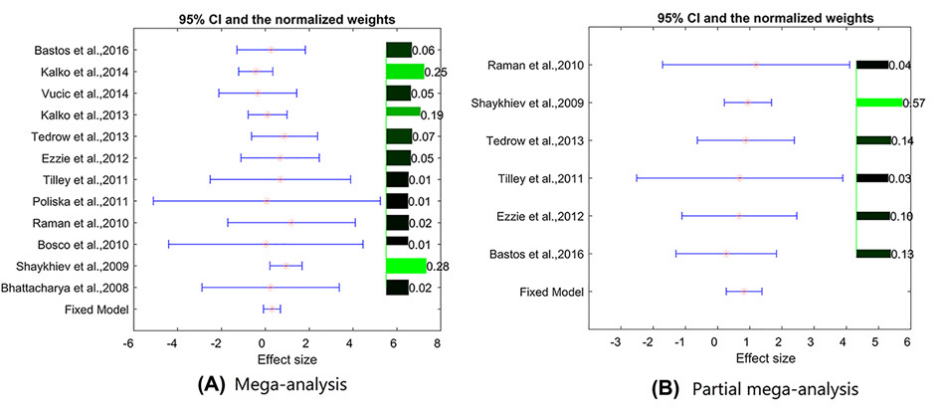
The effect size, 95% confidence interval and weights for the gene *GPNMB* (**A**) Mega-analysis results; (**B**) Partial mega-analysis results. Both results were from fixed-effects model. The bar plot on the right of each figure represents the normalized weights for each dataset/study, ranged within (0, 1); the brighter (green) the color, the bigger the weight (labeled right next to the bar). The star (in red) and lines (in blue) on the left are the mean of effect size (log fold change) and 95% confidence interval (CI) of each dataset/study, respectively.

Heterogeneity analysis indicated no significant between-study variance for both mega-analysis and partial mega-analysis (ISq = 0, *P*-value-*Q* >= 0.69). Therefore, both types of analysis were performed in a fixed-effect mode. For each study, effect sizes, 95% confidence intervals and weights are presented in Figure 2.

In mega-analysis, the expression fold changes of GPNMB were influenced by both population region (country) and sample size (*P*-value = 0.00037 and 0.014, respectively). On the contrary, none of the three parameters (population region, sample size and study date) contributed to the expression levels variance significantly, when only the Top 6 datasets employed in the partial mega-analysis were assessed.

### GSEA analysis results

GSEA process identified 116 GO terms as significantly enriched (*P*<1e-25) by 211 genes implicated with both COPD and OP, which were presented in OP_COPD→GSEA. Table 3 presents the top 10 GO terms with enrichment *P*-value<1.81e-52. Noteworthy, *GPNMB* is an integral component of 17 out of 116 pathways listed at OP_COPD→GSEA, and in one of the Top 10 pathways, namely, one for regulation of cytokine production (Table 3).

**Table 3:** Top 10 GO terms enriched by 211 genes linked to both COPD and OP

GO ID	GO Name	# of Entities	Overlap	*P*-value	Includes GPNMB
0070482	Response to oxygen levels	544	72	7.77e-60	/
0031667	Response to nutrient levels	730	79	7.77e-60	/
0009991	Response to extracellular stimulus	761	79	1.4e-58	/
0001666	Response to hypoxia	424	65	4.69e-58	/
0036293	Response to decreased oxygen levels	461	66	4.04e-57	/
0071407	Cellular response to organic cyclic compound	634	72	1.06e-55	/
0019221	Cytokine-mediated signaling pathway	676	73	4.73e-55	/
0001817	Regulation of cytokine production	789	76	5.79e-54	Yes
0007584	Response to nutrient	370	59	1.76e-53	/
0071396	Cellular response to lipid	767	74	1.81e-52	/

### Pathway Studio-guided analysis of existing literature

**Figure 3:**
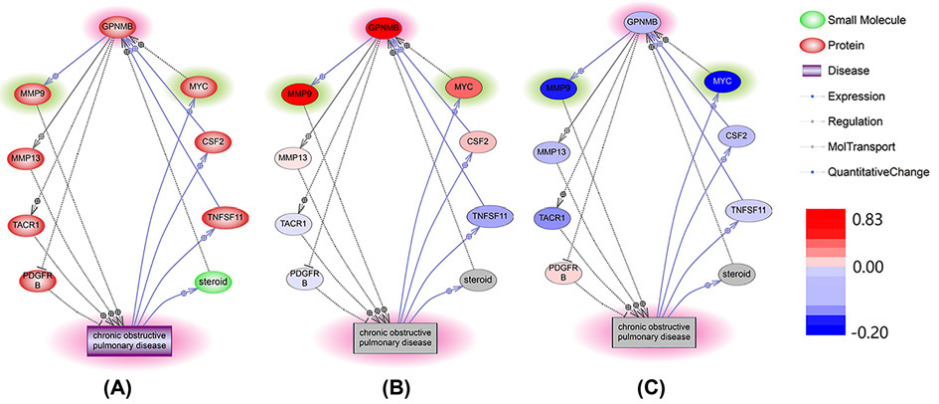
Functional analysis of the molecular pathways connecting COPD and the *GPNMB* (**A**) Pathways linking *GPNMB* and COPD as reconstructed from analysis of previous literature. (**B**) Partial mega-analysis of the expression levels of the genes included in the reconstructed pathways. (**C**) Control mega-analysis of the expression levels of the genes included in the reconstructed pathways performed on datasets excluded from partial mega-analysis. The pathways in (A) was generated in Pathway Studio environment (www.pathwaystudio.com). Each relation (edge) in the figure has one or more supporting references. The colorbar is for the panels (B) and (C) only. Red represents increased expression level, and blue decreased.

According to the *de novo* approach selected for the identification of novel COPD-related genes, no prior direct relations to the pathogenesis of COPD were known for the gene *GPNMB*. To identify connecting genes that link *GPNMB*-encoded molecules to COPD in a unidirectional way, the ‘Shortest Path’ analysis was conducted. As evident from Figure 3A, *GPNMB* may influence the pathogenesis of COPD through multiple pathways. For instance, as knocking down *GPNMB* gene down-regulates the expression of MMP9 [21], and as *GPNMB* directly stimulates expression of MMP9 in fibroblasts [22], one may infer that GPNMB would contribute to the tissue remodeling in COPD airways through promoting secretion of MMP9, which is, indeed, dramatically elevated in COPD [23]. More details of a *GPNMB*→*MMP9*→*COPD* pathway are described in OP_COPD→Pathway_Analysis, including types of the relationship, underlying supporting references, and the related sentences where these relationships have been identified and described.

For the six datasets included in partial mega-analysis, at the level of gene expression, the COPD→MYC→GPNMB→MMP9→COPD circuit has been activated, with an observed expression LFCs of 0.83, 0.80 and 0.35 for *GPNMB, MMP9*, and *MYC*, respectively. In the 9 datasets which did not conform to partial mega-analysis criteria, the same pathway showed as deactivated (averaged LFC<-0.021), mostly liked due to the decreased expression level of *MYC* (LFC = -0.19). At the levels of gene expression performed in COPD samples, other circuits, namely, CSF2 and RANKL/*TNFSF11*, remained deactivated as evident from mega-analysis of both Top 6 and control set of datasets (LFC< 0.085), OP_COPD→Pathway_Mega.

## Discussion

In the present study, we attempted to identify novel, not-yet described molecular connections between OP and COPD. In a large-scale literature lining effort, a total of 210 genes (*P*-value = 2.30e-140) were already highlighted by previous research as commonly involved in both of these conditions, while a total of 371 OP-related genes were prioritized as candidates suitable for further investigation as COPD contributors. In subsequent gene expression mega-analysis, the connections between each of these genes and COPD were tested in 15 COPD related RNA array-expression datasets acquired from GEO (Table 1). While expression levels of 15 genes were significantly changed in COPD as compared with normal controls (*P*-value <0.05), none of these genes satisfied the pre-set significant criteria (*P*<0.005 and effect size (LFC) > 0.59 or < −1). In a partial mega-analysis, which takes into consideration only top performing datasets (*N*=6), a total of 43 OP-related genes were highlighted as displaying significant changes in expression in COPD as well (see in OP_COPD→Partial Mega). Nevertheless, only one OP-related gene, *GPNMB*, passed strict significance of association criteria (Table 2 and Figure 3B). To note, GPNMB didn’t show significance when using 12 studies in the mega-analysis (Figure 3A; LfC = 0.30, and *P*-value = 0.063). MLR analysis showed that sample size and population region (country) were significant influential factors for the expression of GPNMB in COPD, with *P*-values of 0.014 and 0.00037, respectively (OP_COPD→Mega). However, by using partial mega-analysis, it showed that the levels of GPNMB mRNA were increased by more than 50% (LFC = 0.83; *P*-value = 0.0018) in 6 out of 12 studies. This observation identifies GPNMB as a potential COPD biomarker, which awaits further validation.

As shown in Figure 3A, *GPNMB* activity in COPD lungs may be enhanced through many different routes. For example, Granulocyte-macrophage colony stimulating factor (GM-CSF), which is encoded by *CSF2*, is well known as an enhancer of COPD, and even considered as a target for therapeutic neutralization [24]. In fibroblasts, this pleiotropic cytokine directly stimulates expression of *GPNMB* [25,26]. There is another, parallel regulatory circuit that starts with expression levels of transcription factor MYC being required for the activation of the parabronchial smooth muscle cells [27] and dynamically correlated with the pathogenetic progress of COPD [28]. In this loop, MYC upregulates *GPNMB* indirectly, through stimulation of both CSF1 and epICD [29].

To note, some pathways presented in Figure 3A are supported by studies performed in tissues not relevant to studies of COPD. For example, connections between RANKL/*TNFSF11*, a cytokine elevated in COPD [30], and *GPNMB* were studied only in the precursors of osteoclasts. To test the relevance of the seven genes included in Figure 3A, we conducted a mega-analysis for each of them with the datasets included in the partial mega-analysis (Figure 3B) or excluded from this analysis (Figure 3C). Details of this mega-analysis are provided in OP_COPD→ Pathway_Mega.

When *GPNMB* was added to the set of 210 genes previously identified as involved in the pathogenesis of both OP and COPD, a total of 116 enriched pathways were detected (OP_COPD→GSEA) (*P*<1e^−25^). Many of these pathways have obvious connection to both diseases, such as response to hypoxia, which affects the bone marrow stem cells differentiation in bone marrow cavity in osteoporotic patients [31] and induces oxidative stress, coagulation, inflammation, and angiogenesis in COPD [32].

Novel OP-related gene, *GPNMB*, which has been identified as COPD biomarker and COPD pathogenesis contributor in present work, was also an integral part in 17 of these 116 pathways (Table 3). A majority of these enriched pathways are related to the ‘regulation of cytokine production’ theme (GO ID: 0001817), previously implicated in etiology of COPD [11,33,34]. While the roles of *GPNMB* in OP are well-described [35], no apparent connections between this gene and COPD were reported so far. *GPNMB* gene encodes a type 1 transmembrane protein known as glycoprotein non-metastatic melanoma protein B, osteoactivin, dendritic cell-heparin integrin ligand (DC-HIL), or hematopoietic growth factor inducible neurokinin-1 type (HGFIN). Notably, an ectodomain of GPNMB may be cleaved out by MMP-type protease ADAM10, known to promote emphysema of the lungs [36], and shed into circulation as a soluble, biologically active molecule with angiogenic properties [37].

Here, we employed Pathway Studio-guided reconstruction of the pathways through which *GPNMB* could contribute to COPD (Figure 3A). In particular, we identified a vicious circle: COPD→MYC→GPNMB→MMP9→COPD, and, by applying partial mega-analysis technique, showed its consistent activation in COPD-related tissue compartments (Figure 3B). Specifically, COPD progression could lead to significantly increased expression levels of MYC [38]. When combined with the GPNMB→COPD branch [39], MYC up-regulation becomes a part of the vicious circle that prevents COPD patients from achieving resolution of lung inflammation.

Two other reconstructed pathways, GM-CSF/*CSF2* and RANKL/*TNFSF11*, were not supported by partial mega-analysis. This finding may be explained the fact that their sources are outside of the list of tissues typically profiled in COPD-related research. For example, previous studies of osteoclasts precursors exposed to RANKL showed that this cytokine causes hundreds-or even thousands-fold increase in *GPNMB* expression in this type of cells [40,41]. Sheer strength of induced *GPNMB* expression points that its effects may be felt outside of bone compartments, especially given that ectodomain may be easily shed and transported to other tissues. This molecular link may provide possible mechanistic explanation for previously detected systemic increase in the levels of RANKL in patients with COPD [30].

In addition to COPD-related insights, our study also revealed specific strengths and limitations of cross-datasets mega-analysis and partial mega-analysis techniques. As human diseases may differ in the complexity of interplay between various tissues and cells, either the most general (mega-analysis) or more localized (partial mega-analysis) approaches may be found most suitable. Even if it seems that partial mega-analysis may lose at least some of the power of its more generalized counterpart, this methodology allows extraction of additional insights. Closer look at 6 top performing COPD datasets shows that a majority of the samples included in partial mega-analysis were acquired from alveolar macrophages and airway epithelial cells, as well as from the bulk of lung tissue, while the samples excluded from this analysis came predominantly from peripheral blood mononuclear cell (PBMC) and *vastus lateralis* muscle. Even if previous studies have indicated that gene expression signatures in the peripheral blood of patients with COPD do overlap with that of lung tissue or alveolar macrophages [42], and may yield novel, minimally invasive biomarkers for this condition [43], the limitations of these tissue sources are plentiful, with predominant concern of being influenced by a variety of external conditions and treatment modalities [44].

In conclusion, here we present results of systematic, large-scale literature data mining efforts and mega-analysis of gene expression datasets, which allowed us to uncover novel OP-related gene, *GPNMB*, as a previously unrecognized factors to the development of COPD. Analysis of pathway network built upon co-expression of *GPNMB* points that it serves as a bridging factor, which is common for the pathophysiology of OP and COPD, responsive to RANKL and possibly targetable either through RANKL or through MYC, or both.

## Abbreviations

COPD: chronic obstructive pulmonary disease
DC-HIL: dendritic cell-heparin integrin ligand
GSEA: gene set enrichment analysis
HGFIN: hematopoietic growth factor inducible neurokinin-1 type
MLR: multiple linear regression
OP: osteoporosis
PBMC: peripheral blood mononuclear cell
